# Neural Signatures of Hierarchical Linguistic Structures in Second Language Listening Comprehension

**DOI:** 10.1523/ENEURO.0346-22.2023

**Published:** 2023-06-23

**Authors:** Lingxi Lu, Yating Deng, Zhe Xiao, Rong Jiang, Jia-Hong Gao

**Affiliations:** 1 Center for the Cognitive Science of Language, Beijing Language and Culture University, Beijing 100083, China; 2 Center for MRI Research, Academy for Advanced Interdisciplinary Studies, Peking University, Beijing 100871, China; 3 PKU-IDG/McGovern Institute for Brain Research, Peking University, Beijing 100871, China

**Keywords:** EEG, frequency tagging, language proficiency, linguistic structure, neural oscillation, second language

## Abstract

Native speakers excel at parsing continuous speech into smaller elements and entraining their neural activities to the linguistic hierarchy at different levels (e.g., syllables, phrases, and sentences) to achieve speech comprehension. However, how a nonnative brain tracks hierarchical linguistic structures in second language (L2) speech comprehension and whether it relates to top-down attention and language proficiency remains elusive. Here, we applied a frequency-tagging paradigm in human adults and investigated the neural tracking responses to hierarchically organized linguistic structures (i.e., the syllabic rate of 4 Hz, the phrasal rate of 2 Hz, and the sentential rate of 1 Hz) in both first language (L1) and L2 listeners when they attended to a speech stream or ignored it. We revealed disrupted neural responses to higher-order linguistic structures (i.e., phrases and sentences) for L2 listeners in which the phrasal-level tracking was functionally related to an L2 subject’s language proficiency. We also observed less efficient top-down modulation of attention in L2 speech comprehension than in L1 speech comprehension. Our results indicate that the reduced δ-band neuronal oscillations that subserve the internal construction of higher-order linguistic structures may compromise listening comprehension in a nonnative language.

## Significance Statement

Low-frequency neural oscillations are at the root of speech comprehension in a native brain. How a nonnative brain tracks hierarchical linguistic structures in second language (L2) speech and whether it relates to attention and language proficiency has not been established. Our study recorded electrophysiological responses to the linguistic structures at the syllabic, the phrasal, and the sentential rates for L2 listeners and found reduced tracking responses to the higher-order linguistic structures in L2 compared with first language (L1), which relates to L2 proficiency at the behavioral level. Moreover, unlike native listeners, who automatically tracked speech structures without attention, nonnative listeners could not track higher-order linguistic structures in L2 speech during passive listening, indicating a different pattern of attentional modulation in a nonnative brain.

## Introduction

In the era of globalization, it is increasingly important to manage a second language (L2) to facilitate multicultural communication in highly interconnected and diverse human societies. However, people usually find it challenging to comprehend speech in L2 as quickly and as accurately as in their first language (L1), which could be attributed to various sensory and cognitive factors, including less efficient and imprecise auditory encoding, difficulties in speech segmentation, and restricted access to the top-down lexical-semantic system (Flege and Hillenbrand, 1986; Golestani et al., 2009; Mattys et al., 2010; Hervais-Adelman et al., 2014; Lizarazu et al., 2021). Although abundant neuroimaging research has provided valuable insights into the common and distinct neural basis of L1 and L2 processing (Kim et al., 1997; Abutalebi et al., 2001; Perani and Abutalebi, 2005; Tham et al., 2005; Xu et al., 2017), the spectrotemporal features of neural dynamics in L2 comprehension remain unclear because of the low temporal resolution of functional magnetic resonance imaging (fMRI) techniques. Neurophysiological measures such as electroencephalogram (EEG) and magnetoencephalography (MEG) provide a new perspective for this issue by revealing enhanced neural tracking of the speech envelope in L2 listeners relative to L1 listeners (Song and Iverson, 2018; Reetzke et al., 2021), which probably reflects an additional listening effort and cognitive load experienced by L2 listeners acting as a complementary mechanism to overcome L2 comprehension difficulties. Low-frequency neural oscillations are at the root of processing intelligible speech (Zoefel et al., 2018; Etard and Reichenbach, 2019) and have recently been shown to underpin L2 speech acquisition and comprehension (Pérez and Duñabeitia, 2019; Blanco-Elorrieta et al., 2020; Lizarazu et al., 2021). For example, high L2 proficiency was related to stronger δ-band (below 3 Hz) and θ-band (3–8 Hz) neural tracking activity, which corresponded to the phrasal and syllabic rates in natural speech (Lizarazu et al., 2021).

Recently, a novel experimental design for tagging the rhythm of hierarchical linguistic structure in speech has been established (Ding et al., 2016, 2018), which provides compelling evidence that a native brain can parse continuous speech into syllables, phrases, and sentences and concurrently entrain their neural activity to the specific rhythm at different linguistic levels. English listeners who could not understand Chinese failed to track phrases and sentences in Chinese speech, indicating that successful speech comprehension is associated with the internal representation of abstract linguistic structures (Ding et al., 2016). A recent study applying this paradigm in bilinguals showed stronger cortical entrainment to phrases for L2 listeners with high proficiency under a noisy listening environment (Blanco-Elorrieta et al., 2020) but did not report sentential rhythm tracking in either L1 or L2 listeners because of additional environmental noise. Thus, a joint study with multiple linguistic hierarchy levels is needed to provide a more complete view of the neural dynamics underlying L2 speech comprehension by providing evidence on the precise relationship between language proficiency and the neural oscillatory activity that underpins auditory and speech perception.

Whether knowledge-based speech segmentation requires attention is under debate. A line of studies claim that attention and consciousness are required for the knowledge-based organization of speech structures (Makov et al., 2017; Ding et al., 2018), while other studies indicate that higher-order linguistic analysis is maintained in task-irrelevant speech (Gui et al., 2020; Har-Shai Yahav and Zion Golumbic, 2021). In the current study, we broaden this conversation to L2 listening comprehension, with the goals of linking the underlying oscillatory neural representations of hierarchical linguistic structures to language proficiency in L2 listeners and, for the first time, establishing neural evidence for the attentional modulation of speech cortical tracking in L2. Based on previous findings that native speakers can concurrently track hierarchical linguistic structures of speech but that nonnative speakers without lexical-semantic knowledge of the language are unable to track higher-order linguistic structures (Ding et al., 2016) and that L2 listeners have restricted access to the top-down lexical system (Golestani et al., 2009; Mattys et al., 2010), we predicted that nonnative speakers would exhibit reduced neural entrainment to higher-order linguistic structures compared with native speakers. This neural entrainment was expected to be positively associated with L2 proficiency because δ-band tracking is stronger in L2 listeners with a higher proficiency (Lizarazu et al., 2021). Moreover, given that L2 listeners with additional listening effort and cognitive load might differ in their strategy of allocating neural attentional resources to compensate for L2 perception and comprehension difficulties (Song and Iverson, 2018; Reetzke et al., 2021), we anticipated an interaction between attentional modulation (as manipulated with active-listening and passive-listening tasks) and language experience (L1 and L2).

## Materials and Methods

### Participants

The subjects in the L1 group were 24 native Mandarin Chinese-speaking young adults (14 males, 19–31 years old, mean age = 25.3 ± 3.0 years, all right-handed). Subjects in the L2 group were 24 Chinese as a second language (CSL) learners (20 males, 21–31 years old, mean age = 23.7 ± 3.5 years). Nineteen of them were right-handed, and five were left-handed. The L2 participants were international students at Beijing Language and Culture University. Their native languages varied (Extended Data Fig. 1-1), and they had studied Mandarin Chinese as an L2 for one to six years (mean = 3.4 ± 1.6 years). All subjects in the L2 group had passed the Hanyu Shuiping Kaoshi (HSK), with the HSK level at beginner-intermediate Level 3 (*n* = 4)/Level 4 (*n* = 10) to advanced Level 5 (*n* = 4)/Level 6 (*n* = 6). Considering that most of the L2 learners had not been able to take a recent HSK test because of the COVID-19 pandemic, the HSK level might not have accurately reflected the L2 subjects’ actual Chinese proficiency level at the time when the experiment was conducted. To probe this, we set a proficiency test for CSL based on fixed-ratio cloze questions (Feng et al., 2020) shortly before the EEG experiment to allow for an additional evaluation of the participants’ L2 proficiency. This test lasted for 15 min and provided a Chinese proficiency score (0–30) for each L2 subject. The averaged L2 proficiency score for the 24 L2 subjects was 20.2 ± 5.9 (mean ± SD). In the current study, we used the Chinese proficiency score to index L2 proficiency for statistical analysis.

All the participants had normal hearing abilities and had no history of mental disorders, according to their self-reports. They gave written informed consent before participating in the experiment. The study protocol was approved by the Institutional Review Board at Beijing Language and Culture University.

### Stimuli

**Figure 1.**
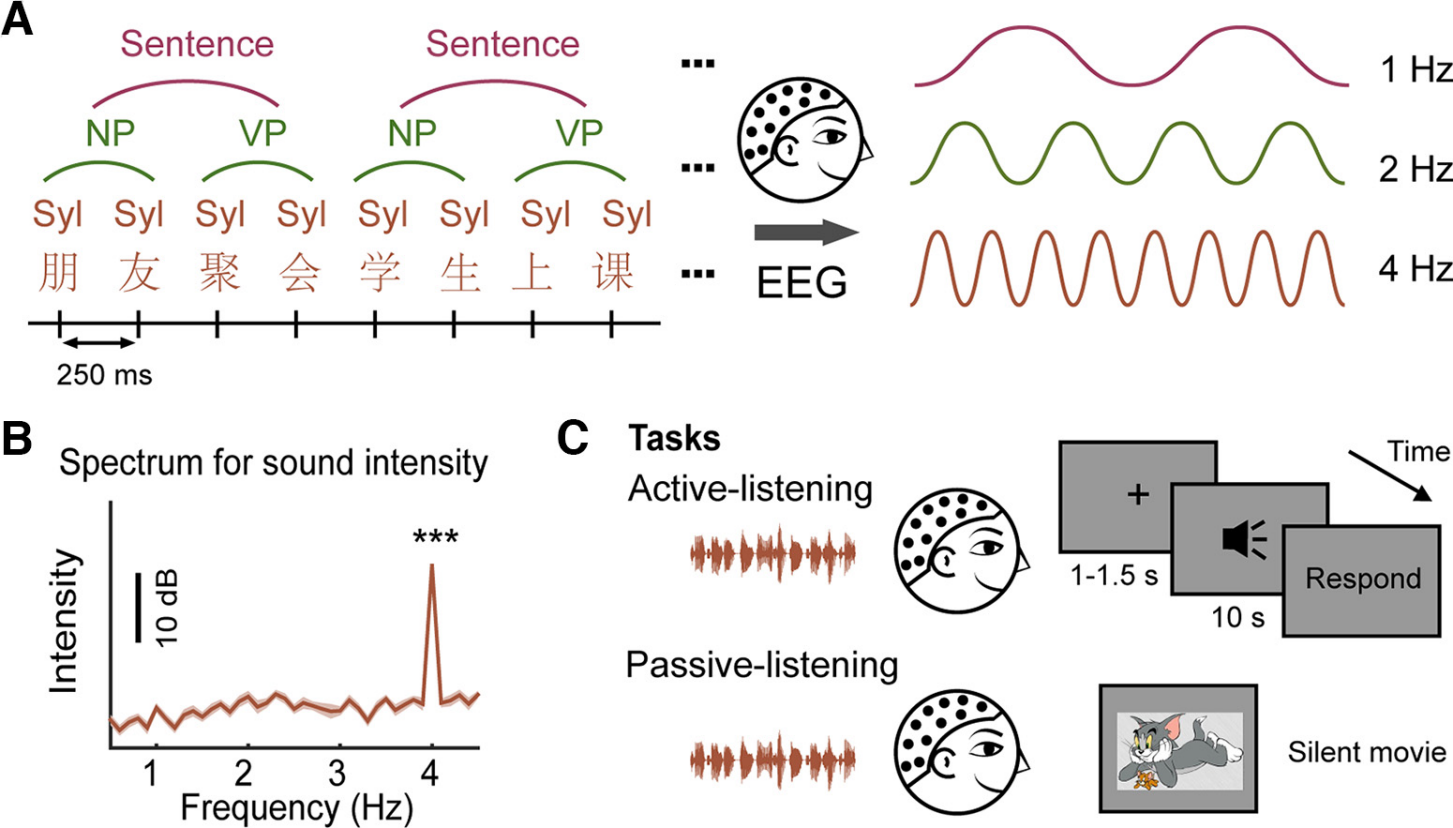
Schematic illustration of the experimental materials and procedures. ***A***, Speech stimuli were sentence sequences. Each sentence consisted of a noun phrase (NP) and a verb phrase (VP), and each phrase contained two 250-ms syllables (Syl). Using EEG, we tested the neural activities at the tagged frequencies of 1, 2, and 4 Hz corresponding to the rhythm of sentences, phrases, and syllables, respectively. ***B***, The spectrum for sound intensity showed a significant peak at 4 Hz (*p* < 0.001), corresponding to the syllable-level fluctuations of the speech. The shaded area represents the standard error (SE) across the 48 speech sequences. ***p < 0.001. ***C***, Experimental protocol. In the active-listening block, participants listened carefully to the speech stimuli and responded to a comprehension question. In the passive-listening block, they concentrated on a silent movie while ignoring the sound. Details of language background of L2 participants are provided in Extended Data Figure 1-1.

A total of 60 sentences were selected from three textbooks for Chinese as second language learners (“Chinese in 10 Days,” “Short-Term Spoken Chinese,” and “HSK Syllabus”). Each sentence was formed by combining a noun phrase (NP) and a verb phrase (VP). Each phrase consisted of two syllables (Fig. 1*A*). The speech stimuli were generated by the neospeech synthesizer using a male speaker (named Liang). The duration of each word was adjusted to 250 ms by padding silence at the end of the audio or truncation at the end with a 25-ms cosine-squared falling ramp. Thus, the sentential, phrasal, and syllabic rates of speech stimuli were tagged at the target frequencies of 1, 2, and 4 Hz, respectively. A total of 48 isochronous speech sequences (duration = 10 s) were generated by randomly choosing 10 different sentences from the 60 sentences. The spectrum of stimulus intensity showed only a spectral peak at 4 Hz among the frequency bins ranging from 0.5 to 4.5 Hz, reflecting the syllabic-rate fluctuations of the speech intensity (Fig. 1*B*). Therefore, we ensured that the cortical tracking of higher-order linguistic structures (i.e., phrase and sentences) was not contaminated by the physical-level sound intensity of the speech stimuli.

10.1523/ENEURO.0346-22.2023.f1-1 Extended Data Figure 1-1 Detailed language background of the 24 subjects in the L2 group. We asked participants to report their native language as well as other languages acquired in addition to their native language and Mandarin Chinese. Participants in the L2 group varied in their native language, which included English (*n* = 7), French (*n* = 5), Nepali (*n* = 4), Samoan (*n* = 4), Spanish (*n* = 1), Urdu (*n* = 1), Chichewa (*n* = 1), and Bengali (*n* = 1). We were primarily interested in participants’ L2 processing of Mandarin Chinese and the comparison of processing between native speakers (the L1 group) and nonnative speakers (the L2 group) that differed in proficiency, regardless of their language background. Thus, the variation in language background did not bias the findings of our study. Download Figure 1-1, DOCX file.

### Procedures

Each subject received an active-listening block and a passive-listening block (Fig. 1*C*). The order of the two blocks was counterbalanced across 24 subjects in each group. Each block contained 48 trials, and the presentation order of the trials was randomized. In the active-listening condition, in each trial, a random silence of 1–1.5 s was followed by the 10-s speech sequence. A fixation cross was shown in the center of the screen. A random interval of 0.5–0.8 s after the sound was terminated, and the question about speech comprehension was displayed on the screen. A five-point scale (1: completely did not understand; 5: completely understand) was also shown on the screen. Participants were instructed to listen carefully to the speech and press a corresponding button on the keyboard after the screen showed the question. After the subject responded, the next trial was presented. The active-listening block lasted for ∼10–12 min, and a short break was arranged after 24 trials to allow participants to rest. In the passive-listening condition, to maintain the participant’s visual attention, we first asked the participant to choose a silent movie without subtitles that they were interested in from some available comedy movies. Then, participants were instructed to concentrate on the movie while ignoring the sound, and they were also warned that they would receive a test about the content of the movie after they watched it. After they started watching the movie, 48 trials of the 10-s sentence sequences were presented, with an interleaved stimulus interval of 1–1.5 s. The passive-listening block lasted for ∼8 min. After the block, we asked the participant to complete a five-point scale about the attractiveness of the movie to them (1: very boring; 3: normal; 5: very attractive).

During the experiment, participants sat in a dimly lit room in front of the monitor. The acoustic signals were digitized at a sampling rate of 22.05 kHz, transferred to a Creative Sound-Blaster X-Fi sound card (Creative Technology Ltd) and delivered bilaterally to subjects via EEG-compatible inserted earphones. The sound pressure was set at a comfortable level and was the same across subjects. An additional questionnaire was presented to L2 listeners after the EEG recording to assess their verbal understanding of the experimental material. Specifically, L2 participants were instructed to answer questions by selecting whether they could recognize each of the 60 verbally printed sentences [the two-alternative forced choice (2AFC)].

### EEG recording and preprocessing

EEG data were collected using a Neuroscan SynAmps 64-channel amplifier (Compumedics). To be consistent with the electrode positions in the 10/20 EEG system, two channels labeled CB1 and CB2 in the 64-channel Neuroscan Quick-cap system were discarded. A total of 62 EEG channels were included in the analysis. The reference electrode was placed on the nose tip, and the impedances of all the Ag/Ag-Cl electrodes were kept below 5 kΩ. Continuous EEG data were recorded at a sampling rate of 1000 Hz and an online bandpass filter of 0.05–400 Hz. The electrooculogram (EOG) was simultaneously recorded by attaching two vertical electrodes upper and lower to the left eye and two horizontal electrodes outer to the canthi of each eye. The artifacts caused by eye blinks and movements were corrected by applying the independent component analysis (ICA) algorithm in the Fieldtrip toolbox (Delorme and Makeig, 2004). Specifically, we calculated the correlation between ICA components and EOG (vertical, the horizontal, and the vector combination of the two) and selected the ICA component(s) with the highest correlation with EOG activity via visual inspection of the topography and time course of the selected component(s) using the Fieldtrip toolbox and homemade codes. The component selection was cross-checked using the Semi-Automatic Selection of Independent Components of the electroencephalogram for Artifact correction (SASICA) plugin in the EEGLAB toolbox (Chaumon et al., 2015). Finally, zero to three ICA components (on average 1.1 components in the L1 group and 1.2 components in the L2 group) were identified and removed from raw data per participant per condition. Then, the data were filtered with an offline bandpass filter of 0.2–60 Hz (fourth order Butterworth IIR filter, two-pass forward and reverse filtered to ensure zero-phase shift) and a notch filter of 50 Hz.

After that, the continuous data were epoched from −1 to 10 s relative to the onset of each speech sequence. After baseline correction, the fast Fourier transform (FFT) was applied to the temporal signal in each 10-s trial, resulting in the frequency resolution of 0.1 Hz. The intertrial phase coherence (ITPC) was calculated among all the trials in the same condition separately for each channel as follows:

$$\text{ITPC}=\left|\frac{1}{n}\overset{n}{\underset{r=1}{\sum }}e^{i\varphi_{r}}\right|,$$

where *n* is the number of trials and φ_r_ is the Fourier phase angle of the stimulus on trial *r*. The ITPC values were *z* score normalized to ITPC_z_ by applying Rayleigh’s Z transformation (Cohen, 2014). An averaged ITPC_z_ across all 62 EEG channels was calculated to represent the individual’s whole-brain cortical tracking of isochronous speech.

### Statistical analysis

In each condition, we examined whether there was a significant response peak at the tagged frequencies of 1, 2, and 4 Hz, as well as the harmonic at 3 Hz. The one-tailed paired-sample *t* test was computed between the ITPC_z_ response at a target frequency bin and the average of its four neighboring frequencies (two neighbors at each side). For example, we compared the peak response at 1 Hz with the average responses at 0.8, 0.9, 1.1, and 1.2 Hz. Here, the null hypothesis was that the spectral response at a target frequency bin was not significantly larger than the average of its neighboring bins. To control for multiple testing, *p* values were adjusted by Bonferroni correction.

When comparing among conditions, we first retrieved the peak value of ITPC_z_ at the target frequencies of 1, 2, and 4 Hz, which reflected the speech cortical tracking to hierarchical linguistic structures of sentences, phrases, and syllables. We conducted ANOVAs on the ITPC_z_ among the between-subject variable of language group (L1 and L2) and within-subject variables of linguistic structures (syllable, phrase, and sentence) and attention (active and passive); the null hypothesis was that neural responses did not differ among conditions. Greenhouse–Geisser correction was applied for violation of sphericity, and Bonferroni correction was applied in *post hoc* pairwise comparisons.

## Results

### Behavioral performance

In the active-listening block, both L1 and L2 listeners reported high speech comprehension scores on the five-point scale (L1 group: 4.96 ± 0.11; L2 group: 4.18 ± 0.47). The independent-sample *t* test showed that the comprehension level in L1 was significantly higher than that in L2 (*t*_(46)_ = 7.844, *p *<* *0.001). In the passive-listening block, participants reported high attractiveness of the movie according to the five-point attractiveness scale in both the L1 group (3.83 ± 1.17, significantly higher than normal level (3), *t*_(24)_ = 3.498, *p *=* *0.002, one-sample *t* test) and the L2 group (4.00 ± 0.83, significantly higher than normal level (3), *t*_(24)_ = 5.874, *p *<* *0.001). There was no significant difference in movie attractiveness between groups (*p *= 0.572). In addition, L2 participants could recognize 88.7 ± 13.7% of the verbally printed sentences in the verbal recognition test, which was significantly correlated with their speech comprehension level in the active-listening task (*r *= 0.574, *p *=* *0.003).

### Frequency-tagged neural responses to speech

**Figure 2.**
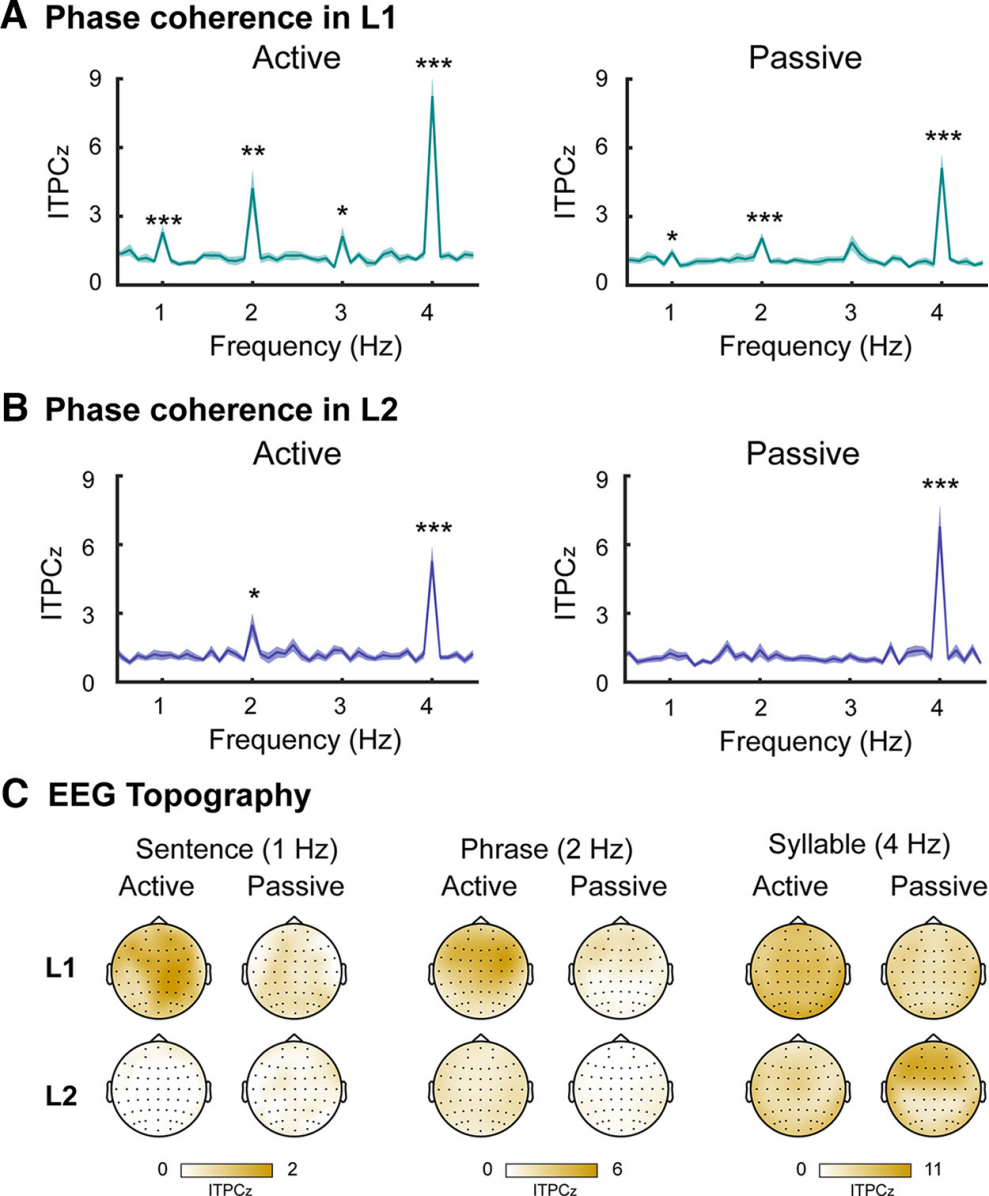
Neural tracking of linguistic structures at syllabic, phrasal, and sentential rates. ***A***, Significant peaks were observed at 1, 2, and 4 Hz in the L1 group, reflecting neural tracking of sentential, phrasal, and syllabic structures in both active-listening and passive-listening conditions. ***B***, In the L2 group, there was robust tracking of the lower-level syllabic-rate fluctuations at 4 Hz and a significant response at the phrasal rate of 2 Hz in the active-listening condition. Shaded areas represent SE; *p <* *0.05, **p <* *0.01, ***p <* *0.001. ***C***, Topographic plots of EEG peak response at tagged frequencies showed whole-brain speech cortical tracking with a general central-frontal distribution. The EEG peak response relative to the average of their four neighboring bins (two at each side) is displayed. There was no difference in hemispheric lateralization between the left-handed and right-handed L2 learners (Extended Data Fig. 2-1).

For L1 listeners (Fig. 2*A*), we found significant peak responses in the active-listening condition at the sentence-level frequency of 1 Hz (*t*_(23)_ = 4.073, corrected *p *<* *0.001), the phrase-level frequency of 2 Hz (*t*_(23)_ = 4.073, corrected *p *=* *0.002) and the syllable-level frequency of 4 Hz (*t*_(23)_ = 7.869, corrected *p *<* *0.001). An additional harmonic was also detected at 3 Hz (*t*_(23)_ = 2.712, corrected *p *= 0.023). In the passive-listening condition, similar neural tracking of linguistic structures was observed at 1 Hz (*t*_(23)_ = 2.667, corrected *p *=* *0.027), at 2 Hz (*t*_(23)_ = 4.483, corrected *p *<* *0.001) and at 4 Hz (*t*_(23)_ = 6.706, corrected *p *<* *0.001). These results indicated that L1 listeners were good at tracking higher-level linguistic structures whenever attention was focused on the auditory stimuli. For L2 learners (Fig. 2*B*), the cortical tracking of higher-level structures was largely reduced, showing only a significant peak at the phrase-level frequency of 2 Hz in the active-listening condition (*t*_(23)_ = 2.926, corrected *p *=* *0.015). Lower-level tracking of syllabic-rate fluctuations was also observed in L2 in both active listening (*t*_(23)_ = 6.464, corrected *p *<* *0.001) and passive listening (*t*_(23)_ = 6.361, corrected *p *<* *0.001). There was no significant difference in hemispheric lateralization between the left-handed L2 learners (*n* = 5) and the right-handed learners (*n* = 19; all *p *>* *0.05, details shown in Extended Data Fig. 2-1). Thus, we plotted the grand averaged EEG topography among subjects in each language group (Fig. 2*C*). The EEG topography displayed whole-brain speech cortical tracking with a general central-frontal distribution, particularly for higher-order linguistic structures.

10.1523/ENEURO.0346-22.2023.f2-1 Extended Data Figure 2-1 Hemisphere lateralization of speech cortical tracking in left-handed and right-handed L2 subjects. Hemisphere lateralization effect was calculated by subtracting the EEG peak response (calculated relative to the average of the four neighboring bins; two on each side) in the left hemisphere from that in the right hemisphere. The hemisphere lateralization effect of the left-handed L2 subjects (*n* = 5) and the right-handed L2 subjects (*n* = 19) in each condition were compared using independent-sample *t* tests. There was no significant difference of hemispheric lateralization between the left-handed and right-handed L2 learners. Download Figure 2-1, DOCX file.

### Language factor interacted with attention to shape speech cortical tracking

To illustrate how language factor and attention interacted in speech cortical tracking, we first conducted an overall language (L1 and L2) by attention (active and passive) by linguistic structure (syllable, phrase, and sentence) three-way ANOVA on the frequency-tagged ITPC_z_. The results showed a significant main effect of language (*F*_(1,46)_ = 5.984, *p *=* *0.018) as well as main effects of attention (*F*_(1,46)_ = 9.203, *p *=* *0.004) and linguistic structures (*F*_(1.702,78.296)_ = 731.203, *p *<* *0.001). The interaction among the three variables was also significant (*F*_(1.699,78.152)_ = 4.380, *p *=* *0.021).

**Figure 3.**
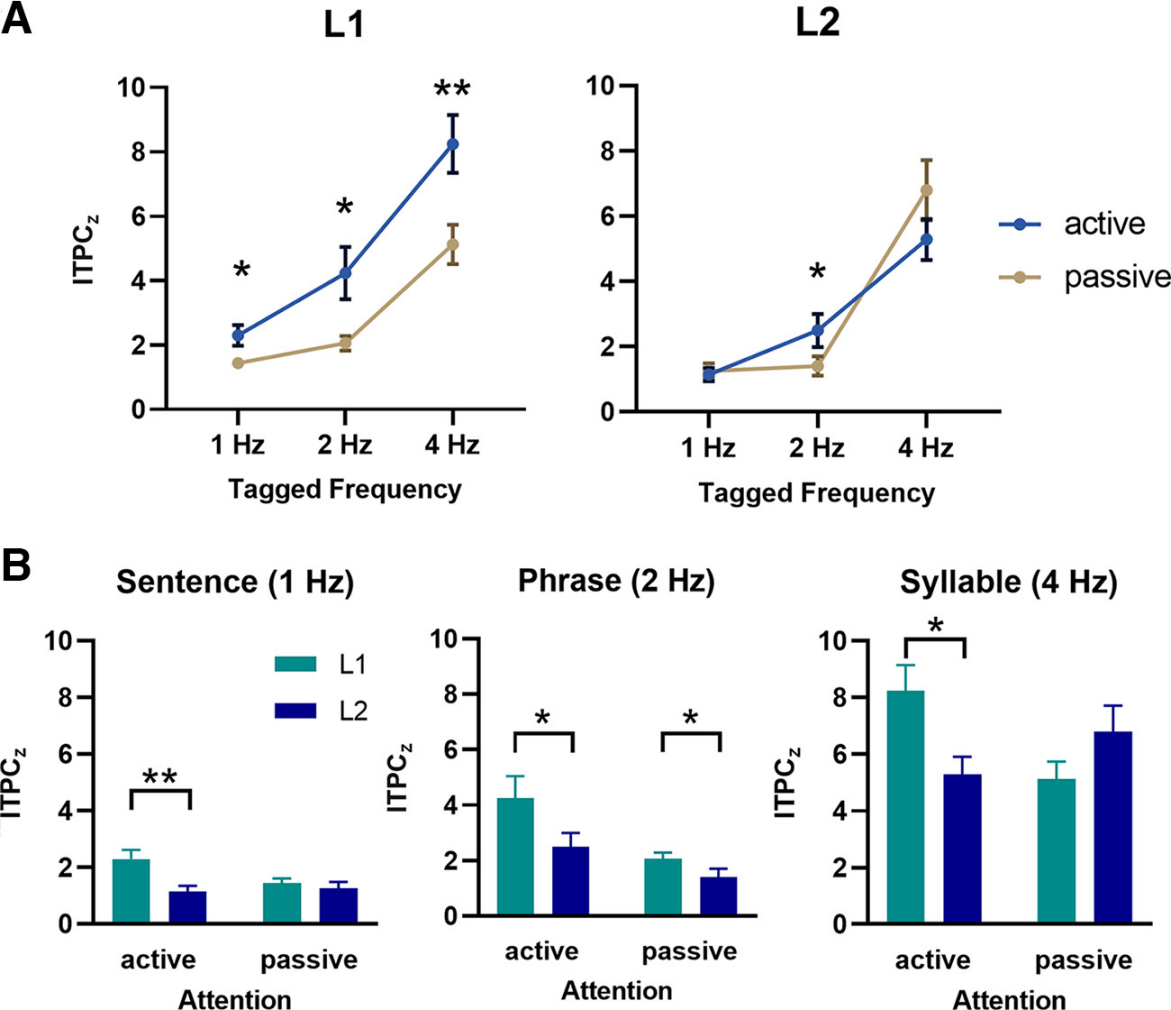
Comparisons of the frequency-tagged neural tracking response between groups. ***A***, The L1 listener’s neural tracking response was stronger in active listening than in passive listening at all tagged frequencies, while the L2 learner’s tracking response was enhanced by attention at a phrasal rate of 2 Hz. ***B***, Neural tracking responses in L2 were weaker than those in L1, especially when attentional resources were allocated to the speech stream. Error bars represent SE; *p <* *0.05, **p <* *0.01.

To untangle the simple effects, we conducted two-way ANOVA on neural tracking responses separately for L1 and L2 (Fig. 3*A*). For L1 listeners, there was a significant main effect of linguistic structure (*F*_(2,46)_ = 32.423, *p *<* *0.001) and attention (*F*_(1,23)_ = 20.053, *p *<* *0.001), with no interaction between them (*F*_(2,46)_ = 2.440, *p *=* *0.098). The neural tracking response was stronger in active listening than in passive listening at the syllable, phrase, and sentence rates (all corrected *p *<* *0.05). However, for L2 listeners, the interaction between attention and linguistic structures reached marginal significance (*F*_(1.387,31.893)_ = 3.692, *p *= 0.051), showing that only at the phrase-level frequency of 2 Hz did L2 listeners take advantage of voluntary attention to enhance their neural responses compared with passive listening (*p *=* *0.029) but not at the other tagged frequencies.

We further conducted language by attention two-way ANOVA at the syllable, phrase, and sentence rates separately to examine the difference between the L1 and L2 groups (Fig. 3*B*). At the syllable rate of 4 Hz, there was a significant interaction between language and attention (*F*_(1,46)_ = 10.637, *p *=* *0.002). Specifically, ITPC_z_ at the syllabic rhythm was lower in L2 than in L1 under the active-listening condition (*p *=* *0.010) but not under the passive-listening condition (*p *=* *0.138). At the phrase rate of 2 Hz, the main effect of language was significant (*F*_(1,46)_ = 4.665, *p *=* *0.036), showing reduced neural entrainment to phrases in L2 than L1. The main effect of attention (*F*_(1,46)_ = 12.517, *p *=* *0.001) was also significant and did not interact with language (*F*_(1,46)_ = 1.388, *p *=* *0.245). At the sentence rate of 1 Hz, the main effect of language was significant (*F*_(1,46)_ = 9.256, *p *=* *0.004). We observed a marginally significant interaction between the two variables (*F*_(1,46)_ = 3.737, *p *= 0.059), indicating a tendency for L2 listeners to have weaker sentence-level tracking than L1 listeners in the active condition (*p *=* *0.004) but not the passive condition (*p *=* *0.138). Overall, cortical tracking of hierarchical linguistic structures was less efficient in L2 than in L1, particularly when attention was allocated to the speech stream.

**Figure 4.**
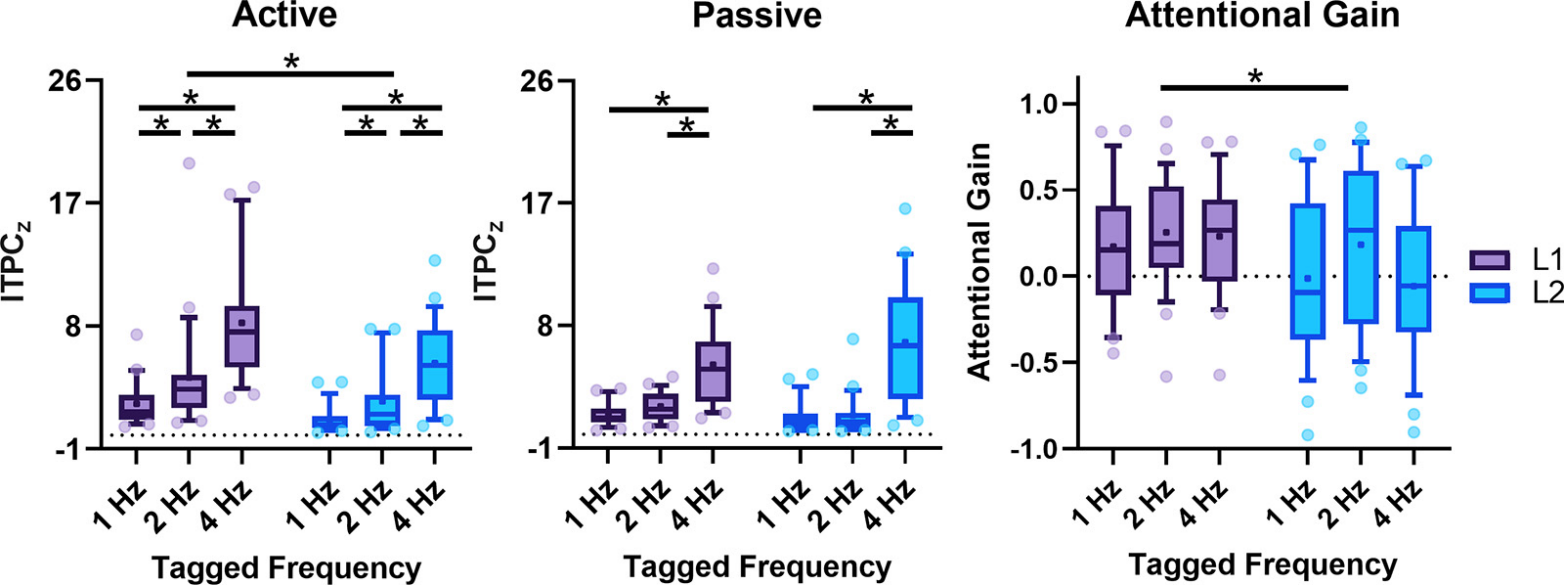
Attentional modulation of speech cortical tracking in L1 and L2. Reduced neural tracking of linguistic structures in L2 relative to L1 was observed in the active-listening condition but not in the passive-listening condition. After normalizing the attentional effect at tagged frequencies, we found less attentional gain in L2 than L1. Data are displayed as box-whisker plots (box, 25/75% percentiles; whisker, 10/90% percentiles; line, median; square dot, mean). Circles indicate values outside the 10th–90th percentile range; * *p *<* *0.05.

In the next step, we looked into how attention modulated cortical tracking responses. As shown in Figure 4, the two-way ANOVA on peak responses found that in the active-listening condition, speech tracking in L2 was weaker than in L1 (*F*_(1,46)_ = 13.743, *p *=* *0.001). Additionally, the main effect of linguistic structures was significant (*F*_(2,92)_ = 37.289, *p *<* *0.001), showing that neural entrainment decreased from lower-level syllables to higher-level sentences (all corrected *p *<* *0.05). Nevertheless, in the passive-listening condition, a significant main effect of the linguistic structure was observed (*F*_(1.268,58.317)_ = 52.612, *p *<* *0.001), reflecting a stronger response at the syllable rate than at the phrase and sentence rate (both corrected *p *<* *0.05), but there was no significant difference between the L1 and L2 listeners when they passively listened to the speech (*p *=* *0.477). Considering that the ITPC_z_ significantly differed among tagged frequencies even in the passive condition, which could be considered a baseline to evaluate the attentional effect, we calculated a normalized attentional gain in each condition to better understand how attention advanced the brain’s neural manifestations across linguistic structures of different levels in L1 and L2.

$$\text{Attentional gain}=\frac{{\text{ITPC}}_{\text{z}}\text{ with attention }–{\text{ ITPC}}_{\text{z}}\text{ without attention }}{{\text{ITPC}}_{\text{z}}\text{ with attention }+{\text{ ITPC}}_{\text{z}}\text{ without attention }}$$

The language (L1, L2) by the linguistic structures (syllable, phrase, sentence) two-way ANOVA on the attentional gain revealed that the main effect of language was significant (*F*_(1,46)_ = 7.839, *p *=* *0.007), showing less attentional gain in L2 than L1. The main effect of the linguistic structure was not significant (*F*_(2,92)_ = 1.734, *p *=* *0.182), nor was the interaction between variables (*F*_(2,92)_ = 0.829, *p *=* *0.440). These data further supported the previous findings of reduced speech tracking in L2 from the perspective that L2 learners’ overall capability of improving speech neural tracking by attention was limited compared with L1 listeners.

### Correlation between language proficiency and cortical tracking of L2 speech

**Figure 5.**
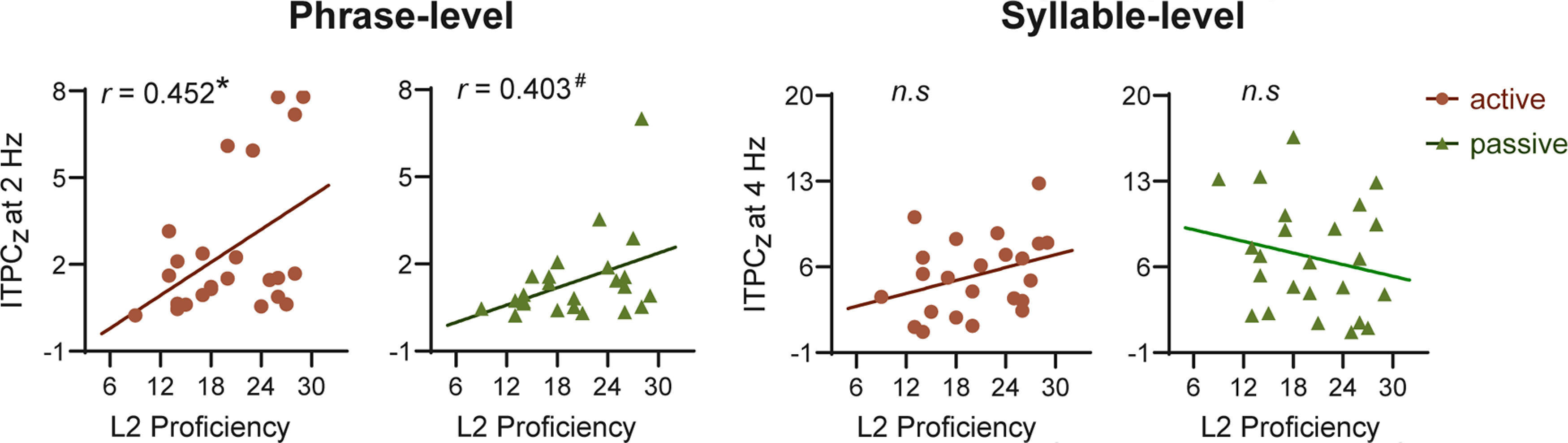
Correlation between language proficiency and cortical tracking of L2 speech. Pearson correlation analysis revealed that the oscillatory neural tracking at the phrase-level frequency (2 Hz) but not at the syllable-level frequency (4 Hz) was correlated with L2 language proficiency. *p <* *0.05, # marginally significant; n.s., not significant.

Having identified the reduced neural tracking of L2 speech, we were interested in the relationship between L2 subjects’ language proficiency at the behavioral level and their neural tracking response at the neural level. We found that ITPC_z_ at the phrase-level frequency of 2 Hz was significantly correlated with the subject’s L2 language proficiency in active listening (*r *=* *0.452, *p *=* *0.026), and the correlation reached marginal significance in passive listening (*r *=* *0.403, *p *=* *0.051), while there was no significant correlation between ITPC_z_ at the syllabic rate of 4 Hz and language proficiency under either active listening (*r *=* *0.335, *p *=* *0.109) or passive listening (*r* = −0.206, *p *=* *0.335) conditions (Fig. 5). In other words, neural tracking of the higher-order linguistic structures (i.e., phrases), but not the lower-level amplitude fluctuation, was functionally associated with L2 language proficiency.

### The effect of the first language of L2 listeners on speech tracking responses

**Figure 6.**
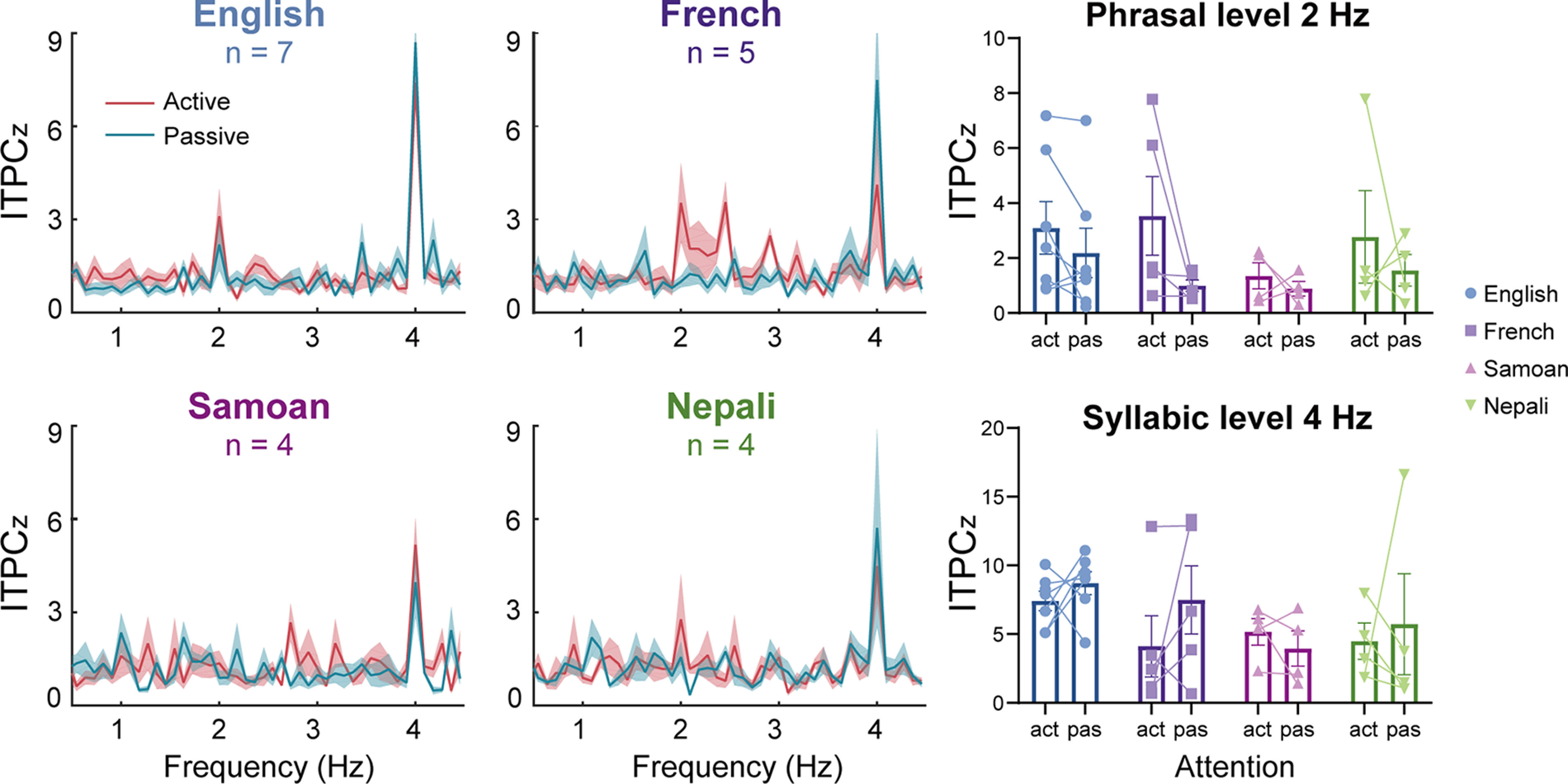
Tracking responses in L2 subgroups whose L1 was English (*n* = 7), French (*n* = 5), Samoan (*n* = 4), or Nepali (*n* = 4). For all subgroups, a paired-sample *t* test showed no significant attentional effect at either 2 or 4 Hz (all *p *>* *0.05). The shaded areas and error bars represent the SE. A connecting line between two dots indicates data from the same individual. act: active; pas: passive.

We divided the L2 data into four subgroups based on listeners’ L1 background: English (*n* = 7), French (*n* = 5), Samoan (*n* = 4), or Nepali (*n* = 4) to explore the possible influence of language background on speech tracking response in L2 listeners. The corresponding spectral response profiles and individual data for each subgroup were displayed in Figure 6, which showed a relatively clear spectral peak at 4 Hz. A two-way language background (English, French, Samoan, Nepali) by attention (active, passive) ANOVA on the 4-Hz peak response showed no significant main effect of language background (*p *=* *0.200) or attention (*p *=* *0.363) or their interaction (*p *=* *0.674). Paired-sample *t* tests on the 4-Hz peak response between active and passive conditions for each subgroup showed no significant attentional effect (all *p *>* *0.05). Additionally, the comparison of the 2 Hz response between the active and passive conditions showed no significant attentional effect in each subgroup (all *p *>* *0.05), although there was a trend of increasing responses when attention shifted from the visual to the auditory modality. Given the small sample size in each subgroup, care should be taken in interpreting the results in the subgroups.

## Discussion

Our study examined the neural tracking response to hierarchical linguistic structure in L1 and L2 speech when participants listened to the speech or ignored it. We found that the native brain reliably tracked the phrasal and sentential rates of speech, and the structure building operation was maintained without auditory attention. For the nonnatives, the neural entrainment to higher-order linguistic structures was markedly reduced when they actively listened to the speech and was eliminated when their attention was distracted. Importantly, we revealed a positive correlation between language proficiency and the neural representation of linguistic structures. In summary, our study not only replicated the neural tracking of hierarchical linguistic structures reported by Ding et al. (2016) but also advanced the current understanding of L2 speech comprehension in two significant ways: describing the low-frequency neural oscillations that concurrently track hierarchical linguistic structures of speech in the brain of a nonnative speaker as well as the effects of top-down modulation of attention and language proficiency on neural linguistic processing.

Consistent with our prediction, we observed robust tracking of hierarchical linguistic structures at 1, 2, and 4 Hz when L1 subjects focused on speech. However, for L2 listeners, the neural tracking at the sentential rate of 1 Hz was fully disrupted, although the L2 listeners had high verbal recognition and auditory comprehension of the speech materials. We propose that the disrupted neural entrainment to the higher-level linguistic structures for L2 listeners could arise from the restricted top-down modulation in speech processing. A number of previous studies have claimed that L2 learners do not anticipate upcoming words to the same extent that native speakers do (Martin et al., 2013; Mitsugi and Macwhinney, 2016; Dijkgraaf et al., 2019; Bovolenta and Marsden, 2022) and that the lexical-semantic analysis of L2 speech at the neural level is significantly delayed compared with L1 speech (Hahne, 2001; Phillips et al., 2006). The reduced neural tracking of L2 speech in our study could be attributed to the less efficient retrieval of top-down linguistic knowledge to assist speech comprehension when the speech was presented with a high speed (i.e., syllabic rate at 4 Hz in the current experiment). Recently, Lizarazu et al. (2021) reported that δ-band neuronal oscillations (below 3 Hz) in higher-order cortical areas that underpin the top-down modulations of the auditory cortex by the inferior frontal and motor cortex decreased for nonnative speakers. Consistent with this observation, our study showed that whole-brain δ-band tracking was reduced at 2 Hz and fully disrupted at 1 Hz for L2 listeners, and δ-band tracking at 2 Hz was functionally associated with L2 language proficiency. Here, we made a step forward by showing that the internal construction of hierarchical linguistic structures may underpin the functional role of δ-band tracking in L2 speech listening comprehension. Limited by the spatial resolution of EEG, we did not localize the neural generators of the δ tracking responses to the higher-level linguistic structures. The neural resources of neural entrainment to speech structures, especially the cross talk between higher-level and lower-level cortical regions, would clearly repay further investigation if techniques with higher spatial resolution were used.

Previous studies have shown that δ-band and θ-band tracking rates are modulated by selective attention while listening to competing talkers (Ding and Simon, 2012; Horton et al., 2013). In our study, a similar attentional effect was found for δ-band tracking (i.e., 1 and 2 Hz), which corresponded to sentential and phrasal structures, and for θ-band tracking (i.e., 4 Hz), which corresponded to syllabic structures in the L1 listeners. In addition, our results support partially automatic tracking of higher-level linguistic structures in a native brain (Gui et al., 2020; Har-Shai Yahav and Zion Golumbic, 2021) by showing that significant δ-band tracking for the phrasal and sentential linguistic structures was maintained when attention was distracted from the visual modality. Note that this result is inconsistent with the finding by Ding et al. (2018) that participants no longer tracked the phrasal structures in speech while watching a silent movie. We believe this inconsistency has two possible explanations. One possibility is that the response phase (i.e., ITPC) might be more sensitive than the response power for detecting the tracking response, as was shown by Ding et al. (2018). The other possibility is that watching silent movies was not a challenging task to distract attention, which might lead to occasional shifts in attention to speech. However, another study by Gui et al. (2020) showed that when applying a more challenging visual task instead of watching a silent movie, native speakers were still able to track the phrasal and sentential rates in speech. In our study, we ensured that attentional resources were distracted to the visual modality by warning the participants that they should prepare for a questionnaire at the end of the movie, and the participants reported the high attractiveness of the movie. Nevertheless, more sophisticated research in the future is expected to address this important issue relevant to our findings by monitoring the behavioral outcomes of the distracting task and manipulating the levels of attentional burden.

Furthermore, we presented a different pattern of attentional modulation for L2 subjects compared with L1 subjects. First, the neural linguistic processing in L2 required top-down attention, as L2 listeners do not track the phrasal rates in speech in the absence of voluntary attention. Second, the attentional gain decreased more in L2 listeners than in L1 listeners regardless of linguistic hierarchy; that is, the neural tracking responses to L2 speech benefited less when attention resources were allocated to the auditory modality than when they were allocated to the visual modality. Notably, in the L1 group, we nicely documented the attentional effect at 4 Hz, as well as at 2 and 1 Hz, while in the L2 group, we found attentional modulation only at 2 Hz (not 4 Hz). The attentive manipulation of syllabic tracking was not successful in the current experiment. The absence of an attentional effect at 4 Hz in L2 could perhaps explained from the perspective of the frequency-tagging paradigm and the experimental task. Previous research has shown that watching a silent movie can impact higher-level linguistic construction (Ding et al., 2018), but it may not be effective in triggering attention-related changes at the acoustic level (Wang et al., 2022). Specifically, syllabic tracking at the acoustic level established by the frequency-tagging paradigm was not sensitive to attention induced by a more demanding visual task (Gui et al., 2020). Given that watching a silent movie was not challenging, it is possible that some L2 subjects may have occasionally shifted their attention to the speech stimuli despite being instructed not to, thereby affecting the syllabic tracking responses at 4 Hz. To further investigate this possibility, future studies should incorporate behavioral measures that accurately assess the attentional state of the participants during EEG recording. Additionally, alternative experimental paradigms, such as interpreting event-related potentials for syllable tracking and using recording techniques with higher signal-to-noise ratio and spatial resolution, such as magnetoencephalography (MEG), should be considered to investigate the attentional effects and explore their underlying neural substrates. Furthermore, conducting channel-of-interest analysis on the auditory electrodes identified by an additional localizer task may also be a valuable approach for future investigations.

In addition, the absence of attentional modulation at 4 Hz in L2 listeners might be attributed to differences in linguistic and acoustic features between their native languages and Mandarin Chinese, which could have resulted in a potential difference in bottom-up attention. For instance, Mandarin Chinese emphasizes a regular syllabic structure by means of its orthography, which can influence speech perception (Ziegler and Ferrand, 1998). It is possible that L2 learners, whose first language lacks such an orthography, have not developed the capacity to suppress such modulatory processes, resulting in increased bottom-up attention when listening to Chinese speech. Moreover, according to the World Atlas of Language Structures (WALS; Dryer and Haspelmath, 2013), there are fundamental linguistic differences between Mandarin Chinese and the native languages of L2 learners, with Mandarin having lower syllable complexity and a higher consonant-vowel ratio than most of L2 listeners’ first languages (e.g., French or English). Low-level acoustic features, such as the sharpness of consonantal beginnings in syllables, affect syllabic-level envelope tracking (Doelling et al., 2014). Thus, it is plausible that L2 listeners’ first language experience influences syllabic-level tracking at 4 Hz, resulting in interacting modulation patterns that contribute to the missing attentional effect.

Finally, as is evident in Figure 5, neural entrainment to phrasal-level structures was associated with L2 language proficiency. These results were consistent with previous findings reporting the modulatory effect of L2 language proficiency on the cortical processing of linguistic information (Liberto et al., 2021) and the relationship between L2 proficiency and low-frequency neural oscillations (Lizarazu et al., 2021). Lizarazu et al. (2021) pointed out that both δ-band tracking for phrases (below 3 Hz) and θ-band tracking for syllables (3–8 Hz) were related to L2 learning proficiency. However, in our study, we revealed a significant correlation between L2 proficiency and phrasal-level tracking at 2 Hz but not with syllabic-level tracking at 4 Hz. The inconsistency may arise from the frequency-tagging paradigm that we applied in our study, which captured neural oscillatory signals concentrated at a single frequency bin (i.e., 4 Hz) instead of a broadband range. Based on our findings, we proposed that neural entrainment to higher-order linguistic structures (i.e., phrases) is functionally related to L2 language proficiency.

There are some limitations of the current study. First, we did not strictly control the L1 background of L2 subjects. Although including a variety of native languages in the L2 group enabled us to generalize the results beyond a specific native language, we urge caution in interpreting the results from the perspective of the L1–L2 interaction. We performed a preliminary analysis of the speech tracking response in nonnative listeners with the same L1 background (Fig. 6), which was limited by the small sample size in each subgroup. Future research with larger sample sizes and increased statistical power will for allow more representative sampling and thorough investigation of how the listener’s L1 experience (for example, with different preferred word orders) influences L2 speech comprehension. Second, we adopted a previously developed paradigm of concurrent neural tracking (Ding et al., 2016, 2017, 2018; Makov et al., 2017; Lu et al., 2019, 2021; Blanco-Elorrieta et al., 2020), in which the neural responses to hierarchical linguistic structures were simultaneously tagged at different frequencies. Since this paradigm risks contamination from harmonics on the tracking responses, separate conditions of linguistic structures (Sheng et al., 2019; Gui et al., 2020) should be considered in future neural tracking studies.

In summary, the present findings highlight the neural signatures of hierarchical linguistic structures (syllables, phrases, and sentences) in L2 speech comprehension by revealing disrupted neural oscillations entrained to higher-order linguistic structures in a nonnative brain compared with a native brain. Importantly, this work discloses a more complex and informative pattern than was previously known regarding how attention and language proficiency modulate L2 speech tracking and reveals a neurophysiological manifestation of speech structure construction that may underlie the pervasively experienced phenomenon of compromised listening comprehension in a nonnative language.

## Synthesis

Reviewing Editor: Anne Keitel, University of Dundee Decisions are customarily a result of the Reviewing Editor and the peer reviewers coming together and discussing their recommendations until a consensus is reached. When revisions are invited, a fact-based synthesis statement explaining their decision and outlining what is needed to prepare a revision will be listed below. The following reviewer(s) agreed to reveal their identity: Alessandro Tavano, Mathias Scharinger.

The reviewers and editor felt that, while most comments have been satisfactorily addressed in the response letter and revised manuscript, the main issue of the missing attention effect needs further analyses.

During joint discussion between both reviewers and editor, the following suggestions were made to attempt to clarify why there is no expected, low-level attention effect at 4 Hz in L2 listeners:

“It would be informative to split the data either time-wise or subject-wise. Time-wise, it would be interesting to see the first 24 items in a block compared to the last 24 items (split-half analysis). Thereby, it might be possible to see whether the lack of the attention effect results from a decline during the task. A subject-wise split would be to look at subgroups of L2, determined by their L1. Here I would like to suggest to look at English and French participants (L1) separately. Of course, the two split procedures can be combined. In total, the authors could thereby attempt to find possible explanations for the lack of the attention effect.”

In addition, Reviewer #1 detailed their request further during the discussion session:

“While I appreciate the efforts of the authors in rewording their paper away from the attention effect as a central feature, it is nevertheless still part of the conclusions in the Abstract and Significance Statement. It has not been cut out.

Rewording the paper did not comply with the request that the issue be “thoroughly addressed in the revised version of the manuscript”.

In my view, this is such a basic issue that it must either be met with clear explanations or be completely cut out of the paper. No third way, that is rewording.

The authors’ argument relies on the fact that:

1. Attention away from stimuli did not lead to a difference between L1 and L2 at 4 Hz (syllable tracking).

2. Attention to stimuli increased tracking for L1 but not L2.

Hence, they conclude that L2 listeners likely did not covertly pay attention to stimuli when required not to. This is a fair conclusion, in my opinion.

However, they add that the absence of an attention effect for L2 is likely due to a weaker attentive syllabic tracking in L2.

However, this is not acceptable as an explanation.

If it does not stem from a technical issue, this finding points at an interaction issue between attention and familiarity.

Namely, that one could not have basic attention effects without familiarity.

This would be at odds with the conceptual distinction the authors rely on (and is generally assumed in the literature) between attention as a domain-general

capacity (enhancing acoustic tracking, in this case) and familiarity as a top-down mechanism based on expectancies.

I want to know which is which: is it a technical issue, or should the authors conclude that attention to speech is inherently a top-down effect (thus changing the layout of their paper/conclusions)?

To verify this, the authors must do the following, including a full report in their reply:

1. Verify the performance of their ICA decomposition. It is not OK to use a 0.2 HP filter for ICA because it does not help with data stationarity (long drifts remain in the data).

Please try redoing the ICA with 1 Hz HP. It is not OK to LP filter to 60 Hz, better to about 100 Hz, in order to keep the very fast components of eye movements inside the data, so that the algorithm can model those.

2. How many EOG (vertical/blink, horizontal) ICA components were found per participant? Please report group range.

3. Please use SASICA or MARA or comparable semi-automatic toolboxes to classify and exclude ICs which can lead to a reduction in SNR (muscle, line noise etc.). Again, how many of each type in each group.

4. Please report the length of the filter and the size of the transition band.

5. Please consider an analysis focused on auditory electrodes (e.g., if no auditory localize was used, the ones with top quartile phase coherence when all conditions are averaged toghether), instead of an average across 62 EEG electrodes. There could be more task-unrelated noise in the L2 attentive condition (say, in visual areas), leading to reduced overall phase coherence.”

Please see below for detailed, unabridged comments made by the reviewers. Please respond to the above questions, as well as to the below comments.

*****

Reviewer #1 - Advances the Field

I think this paper is potentially very interesting, but the revised paper fails short of providing the required answers.

Reviewer #1 - Comments to the Authors

The main concern about the absence of a difference between active and passive conditions has not been satisfactorily addressed.

In their rebuttal letter, the authors initially argue for the use of a silent movie as a common strategy in determining a pre-attentive (not really passive) listening condition. That is obviously fine, but redundant, as it begs the original question without answering it, as in all the cited literature the silent video strategy succeeds in establishing a significant difference in the dependent measure of choice for the attention effects.

I understand that the authors consider the absence of a difference between L1 and L2 in the passive (pre-attentive) condition, and its presence in the attention condition a sufficient argument to conclude that L2 listeners where “unlikely to pay more attention to speech under passive condition compared with L1 listeners”, but this again begs the question that there must be a significant difference between passive and active conditions to speak of an attention effect. Otherwise, one cannot speak of an attention effect, simply put.

Reasons may vary: low SNR in some participants, low adherence to instructions by some participants. I cannot answer this issue, only the authors can, by profoundly revising their analysis pipeline, and finding the locus of the problem.

One cannot accept the Liu et al. (2022) as a justification, because the authors did not test the trade-off between lower-acoustic and higher-linguistic processes.

The authors’ comments about the diverse linguistic background are not fully convincing, but there the issue is - I think - easier to solve, by providing analyses of more homogeneous subgroups of participants. I would be convinced if for example the French vs Nepali vs Samoan subgroups - which are equally powered - would show a similar spectral response profile.

*****

Reviewer #2 - Advances the Field

See previous comments - the very good revisions warrant an advancement for the field.

Reviewer #2 - Comments to the Authors

Thank you for the careful revision and detailed responses to the comments of my review from the first round. While I still think it would be interesting to learn more about possible tracking differences on the basis of L1 (e.g. by looking at the largest two L1 populations English and French), I understand that the design of the study does not allow a statistically robust analysis in this regard. All other responses are satisfying and comprehensible.

## Author Response

Synthesis of Reviews:

Synthesis Statement for Author (Required):

The reviewers and editor commended the work done by the authors in this revision. Still, the following main points can be identified from the reviewers’ comments:

(1) The presented analyses highlight the need to phrase any conclusions very carefully.

(2) The lack of an attention effect at 4 Hz deserves a thorough discussion.

(3) The additional analysis of L1 groups should be included in the manuscript.

You will find these main issues as well as other comments in the unabridged reviewer comments below. Please address all comments in a point-by-point manner.

We sincerely appreciate the time that you and the reviewers have taken to provide constructive feedback to help improve the quality of our manuscript. We have thoroughly addressed these points in this revision, and the detailed responses are provided in the following reply.

Reviewer #1

Advances the Field (Required)

The main point of the experiment (attentive manipulation) did not work. Hence, there is no real advancement. However, there are interesting bits and pieces which could warrant publication at the Editor’s discretion.

Thank you for taking the time to review our paper and providing valuable feedback. Our research has uncovered significant findings regarding non-native listeners’ ability to track higher-order linguistic structures in L2 speech, as well as its relationship with L2 proficiency. We believe that our findings will be of interest to the neuroscience community and provide guidance for future explorations on this topic.

Comments to the Authors (Required)

Time effect on attention, first vs last trials.

Since the data distribution in their very small sample size is unlikely to fit the assumptions of parametric tests, likely leading to non-significant findings, and since the absence of a significant effect is beneficial to the authors’s position, scientifically their conclusions area dubious.

Group split by L1 background

Same as above, only that this time is goes against the authors’s main conclusions in the paper, so they highlight the difficulty of drawing valid conclusions. But then, the same should be said for the time effect comparison.

When conduct time-wise splitting of the data, we calculated ITPC values form the first 24 trials and the last 24 trials. Though the reduction of trial number may lead to decreased SNR for each subject, the group-level sample size was still n = 24. We did not find any significant decline in the attention effect over time as previously reported.

Regarding subject-wise splitting of the data, there were no significant differences in the attentional effect among different groups based on native language. However, it is worth noting that the sample size entered into the group-level statistical analysis was reduced to n = 7 (English), n = 5 (French), n = 4 (Samoan), and n = 4 (Nepali). The statistical results are therefore limited by the small sample size.

The main point we want to emphasize is that after examining both the time and group effects, as well as their combinition, no significant effects were found. We hope this clears up any concerns raised in this comment.

Figure 2, syllabic tracking effects in small sample sizes are to be expected, as it is usually present at individual subject level.

With a small sample size, we observed spectral peaks at the syllabic rate of 4 Hz that was present in individual subjects (see new Figure 6 in this revision). However, upon examining the attention effect at 4 Hz (by comparing the active condition with the passive condition), we did not observe a significant attentional effect.

I agree with the authors statement that “The key point we would like to emphasize is that while higher-level linguistic processing is sensitive to a shift of attention from the auditory to the visual modality, the syllabic tracking response at the acoustic level, as measured with the frequency-tagging paradigm, displayed relatively low sensitivity to a visual distractor.” Thus, they should conclude that the attentive manipulation of speech tracking was not successful in this experiment (for whatever reason, as the ones put forward are tentative).

As per the suggestion, we have added a statement to acknowledge that the attentive manipulation of syllabic tracking was not successful in the current experiment (line 378-379)

Hence, in their ms they should provide possible explanations in a tentative working: not “The absence of an attentional effect at 4 Hz in L2 can be explained”, but “The absence of an attentional effect at 4 Hz in L2 could perhaps be explained”, or similar.

As per the suggestion, we have rewriten this sentence with a tentative expression (line 380).

The authors should include the reanalysis of homogeneous L1 groups in the main paper. It is a nice addition, and it was quite some work, so it should be highlighted.

Following the suggestion, we have included the analysis of L2 subgroups based on L1 in the main content of this revision (line 298-311, Figure 6).

Reviewer #2

Advances the Field (Required)

The authors present a very interesting and timely study on the neural mechanisms of processing a second language. The manuscript has improved compared to the initial submission.

We thank the reviewer for taking the time to review our manuscript and providing insightful comments to improve the quality of our work.

Comments to the Authors (Required)

Summary

----------------------------

The authors have resubmitted a study on cortical tracking of speech at different functionally relevant frequencies in a second language (L2) compared to native language (L1). They rely on a previously used frequency-tagging design developed by Ding and colleagues and crucially extend this line of research to second-language processing. Additionally, the author vary attention and thereby include the attentional modulation of cortical tracking as has been done in studies concerned with the “Cocktail party” situation (i.e., selective attentional focus on a speaker in multiple speaker noise).

The results indicate a disruption of tracking at lower (sentence-related) frequencies for L2 listeners. L2 listeners furthermore showed a correlation between tracking at sentence-level frequencies and proficiency. Finally, top-down modulation of attention was less efficient in L2 compared to L1.

We thank the reviewer for the excellent summary of our work.

Overall assessment

----------------------------

The authors present a very interesting and timely study on the neural mechanisms of processing a second language. The manuscript has improved compared to the initial submission. However, the lack of attentional modulation at 4Hz for L2 speakers could still be explored in more detail (see below)

We appreciate the positive feedback. Based on the reviewer’s suggestion, we have included a new discussion regarding the lack of attentional effect at 4 Hz for L2, which is detailed in the following section.

Major concern

----------------------------

As mentioned by both reviews on the basis of the first submission, the lack of attentional modulation at 4 Hz deserves thorough discussion. The authors have now included some more background data regarding the first language of the L2-speakers. Of course, due to the small and not controlled sample-sizes, these additional data are to be taken with care. However, the authors could still discuss a possible “bottom-up attentional” difference, that is rooted in the prosodic/syllabic material of the respective languages. The idea is that due to the first language of the L2-participants, low-level features of the material caught attention in a bottom-up way, alleviating the experimentally induced top-down attentional modulation. Why should this be?

First of all, Chinese (Mandarin Chinese) emphasises a (regular) syllabic structure by means of its orthography. Since the influence of orthography on auditory processing is quite strong and established (e.g., Ziegler, J. C., & Ferrand, L. (1998). Orthography shapes the perception of speech: The consistency effect in auditory word recognition. Psychonomic Bulletin and Review, 5, 683-689.), it is possible that learners of Chinese have not yet acquired a potential mechanism to suppress such modulatory processes while listening to speech. Another possible source for enhanced bottom-up attention may have to do with the differences in syllable complexity in Mandarin Chinese and (most of) the first languages of the L2 speakers (German, French, English..). Based on typological data, syllables in Chinese show a lower degree of complexity than in French or English (https://wals.info/feature/12A#2/19.3/152.9). Would the authors deem it possible that syllable complexity might modulate syllabic tracking, or, for the purpose of explaining the lack of attentional modulation, cause interacting modulation patterns, depending on L1? If tracking is based on the sharpness of consonantal beginnings in syllables (as suggested by Doelling, K. B., Arnal, L. H., Ghitza, O., & Poeppel, D. (2014). Acoustic landmarks drive delta-theta oscillations to enable speech comprehension by facilitating perceptual parsing. Neuroimage, 85, Part 2, 761-768), the consonant-vowel ratio of the native languages might also influence the parsing of Chinese (with a relatively average consonant-vowel ratio, see https://wals.info/feature/3A#2/19.3/152.9). Altogether, a short discussion along these lines would make the current study stronger, in my opinion.

We greatly appreciate the reviewer’s constructive suggestion. We agree with the reviewer’s interpretation that the lack of attentional modulation at 4 Hz in L2 listeners might be due to differences in linguistic and acoustic features between their native languages and Mandarin Chinese, leading to potential variations in bottom-up attention. Following the suggestion, we have added a new discussion in this revision to address this issue in greater details (line 396-409).
