# Supplementary material for: Neural Signatures of Hierarchical Linguistic Structures in Second Language Listening Comprehension
Source: eNeuro. 2023 Jun 23;10(6):ENEURO.0346-22.2023. doi: 10.1523/ENEURO.0346-22.2023 (PMC10294774; doi:10.1523/ENEURO.0346-22.2023)
Supplement: Extended Data Figure 1-1 — Detailed language background of the 24 subjects in the L2 group. We asked participants to report their native language as well as other languages acquired in addition to their native language and Mandarin Chinese. Participants in the L2 group varied in their native language, which included English (n = 7), French (n = 5), Nepali (n = 4), Samoan (n = 4), Spanish (n = 1), Urdu (n = 1), Chichewa (n = 1), and Bengali (n = 1). We were primarily interested in participants’ L2 processing of Mandarin Chinese and the comparison of processing between native speakers (the L1 group) and nonnative speakers (the L2 group) that differed in proficiency, regardless of their language background. Thus, the variation in language background did not bias the findings of our study. Download Figure 1-1, DOCX file. [file enu-eN-NWR-0346-22-s02.docx]

**Extended Data**

**Figure 1-1. Detailed language background of the 24 subjects in the L2 group.** We asked participants to report their native language as well as other languages acquired in addition to their native language and Mandarin Chinese. Participants in the L2 group varied in their native language, which included English (n = 7), French (n = 5), Nepali (n = 4), Samoan (n =4), Spanish (n = 1), Urdu (n = 1), Chichewa (n = 1) and Bengali (n = 1). We were primarily interested in participants’ L2 processing of Mandarin Chinese and the comparison of processing between native speakers (the L1 group) and nonnative speakers (the L2 group) that differed in proficiency, regardless of their language background. Thus, the variation in language background did not contaminate the findings of our study.

| Subject  No. | Native language | Other languages (excluding native language and Chinese) |
| --- | --- | --- |
|  |  |  |
| 1 | English | Shona |
| 2 | English | Hague |
| 3 | English | Spanish, Creole |
| 4 | English | Creole |
| 5 | English | Setswana |
| 6 | English | - |
| 7 | English | - |
| 8 | French | English |
| 9 | French | English |
| 10 | French | English, Spanish |
| 11 | French | English, , Fongbe |
| 12 | French | Songhay, Tamasheq, English |
| 13 | Nepali | Hindi, English |
| 14 | Nepali | Hindi, English |
| 15 | Nepali | Hindi, English |
| 16 | Nepali | English, Japanese |
| 17 | Samoan | English |
| 18 | Samoan | English |
| 19 | Samoan | English |
| 20 | Samoan | English |
| 21 | Spanish | English |
| 22 | Urdu | English |
| 23 | Chichewa | English |
| 24 | Bengali | English, Hindi |
