# Supplementary material for: Neural Signatures of Hierarchical Linguistic Structures in Second Language Listening Comprehension
Source: eNeuro. 2023 Jun 23;10(6):ENEURO.0346-22.2023. doi: 10.1523/ENEURO.0346-22.2023 (PMC10294774; doi:10.1523/ENEURO.0346-22.2023)
Supplement: Extended Data Figure 2-1 — Hemisphere lateralization of speech cortical tracking in left-handed and right-handed L2 subjects. Hemisphere lateralization effect was calculated by subtracting the EEG peak response (calculated relative to the average of the four neighboring bins; two on each side) in the left hemisphere from that in the right hemisphere. The hemisphere lateralization effect of the left-handed L2 subjects (n = 5) and the right-handed L2 subjects (n = 19) in each condition were compared using independent-sample t tests. There was no significant difference of hemispheric lateralization between the left-handed and right-handed L2 learners. Download Figure 2-1, DOCX file. [file enu-eN-NWR-0346-22-s03.docx]

**Extended Data**

**Figure 2-1. Hemisphere lateralization in speech cortical tracking for left-handed and right-handed L2 subjects.** Hemisphere lateralization effect was calculated by subtracting EEG peak response relative to the average of their four neighboring bins (two at each side) in the left hemisphere from that in the right hemisphere. The hemisphere lateralization effect of the left-handed L2 subjects (n = 5) and the right-handed L2 subjects (n = 19) in each condition were compared using independent *t* tests. There was no significant difference of hemispheric lateralization between the left-handed and right-handed L2 learners.

| Condition | Freq. | Left-handed  (M ± SD) | Right-handed  (M ± SD) | Statistical test | | |
| --- | --- | --- | --- | --- | --- | --- |
|  |  |  |  | T value | df | P value |
| Active | 4 Hz | 0.17 ± 1.46 | -0.39 ± 2.06 | 0.57 | 23 | 0.58 |
|  | 2 Hz | 0.20 ± 1.03 | 0.11 ± 0.63 | 0.26 | 23 | 0.79 |
|  | 1 Hz | 0.19 ± 0.98 | -0.08 ± 0.48 | 0.87 | 23 | 0.39 |
| Passive | 4 Hz | -0.66 ± 1.93 | -0.70 ± 2.25 | 0.04 | 23 | 0.96 |
|  | 2 Hz | 0.09 ± 0.31 | 0.23 ± 0.38 | -0.75 | 23 | 0.46 |
|  | 1 Hz | 0.00 ± 0.65 | 0.04 ± 0.49 | -0.17 | 23 | 0.87 |
